# Terminally exhausted CD8^+^ T cells in solid tumors: biology, biomarker potential and translational tools for precision oncology

**DOI:** 10.3389/fimmu.2025.1709852

**Published:** 2026-01-12

**Authors:** Xuejun Guo, Shuhan Ma, Jingwen Wang, Yilin Fu, Wenxue Ma

**Affiliations:** 1 Department of Hematology, Puyang Oilfield General Hospital Affiliated to Henan Medical University, Puyang, Henan, China; 2 Puyang Cell Therapy Engineering Technology Research Center, Puyang, Henan, China; 3 Sanford Stem Cell Institute, Department of Medicine, Moores Cancer Center, China Medical University-the Queen’s University of Belfast Joint College, Shenyang, Liaoning, China; 4 Sanford Stem Cell Institute, Department of Medicine, Moores Cancer Center, University of California San Diego, La Jolla, CA, United States

**Keywords:** digital pathology, immunotherapy biomarkers, precision oncology, T cell exhaustion, terminally exhausted CD8⁺ T cells, tumor microenvironment

## Abstract

Terminally exhausted CD8^+^ T cells (Ttex) are emerging as clinically relevant immune subsets across solid tumors, marked by sustained inhibitory receptor expression, loss of TCF1, and limited proliferative capacity. Once considered functionally inert, Ttex are now recognized for their residual cytotoxic potential and strong associations with tumor immunogenicity, including microsatellite instability (MSI), high tumor mutational burden (TMB), and neoantigen load. Importantly, the prognostic significance of Ttex is highly tumor-context-dependent, shaped by stromal architecture, mutational burden, and progenitor Tpex availability. This review examines the biology, spatial localization, and prognostic value of Ttex, highlighting the Ttex/CD8^+^ ratio as a promising biomarker in cancers such as colorectal, lung, and esophageal carcinoma. We summarize recent advances in multiplex imaging, digital pathology, and AI-driven quantification that support the clinical integration of Ttex assessment. In addition, we discuss emerging therapeutic strategies targeting Ttex through immune checkpoint combinations, thymocyte selection-associated high mobility group box protein (TOX) and circRNA-mediated reprogramming, and exhaustion-resistant T cell engineering. Finally, we outline translational priorities including assay harmonization, functional validation, and longitudinal profiling to advance Ttex-based precision oncology.

## Highlights

- Ttex cells reflect chronic antigen stimulation and advanced exhaustion.
- Ttex/CD8^+^ ratio serves as a tumor-context–dependent prognostic biomarker.
- MSI and TMB are strongly associated with Ttex enrichment.
- Spatial and digital pathology enable standardized Ttex quantification.
- Ttex metrics inform emerging immunotherapies and combination strategies.

## 1 Introduction

Cytotoxic CD8^+^ T cells are central to the immune system’s ability to eliminate malignant cells and are a cornerstone of successful anti-tumor immunity (1, 2). However, in the setting of chronic antigen stimulation such as within the tumor microenvironment (TME) of solid tumors these cells progressively lose effector function, a process known as T cell exhaustion (3, 4). While T cell exhaustion was initially viewed as a uniform state of dysfunction, it is now recognized as a heterogeneous and dynamic spectrum ranging from progenitor exhausted (Tpex) to terminally exhausted (Ttex) phenotypes, each with distinct functional, transcriptional, and spatial profiles (5).

Ttex often defined by high expression of inhibitory receptors [e.g., programmed cell death protein 1 (PD-1), T-cell immunoglobulin and mucin domain-containing protein 3 (TIM-3)] and loss of proliferative capacity [e.g., transcription factor 1 (TCF1^-^)], represent a late-stage differentiation state (6, 7). Though functionally impaired, Ttex cells retain partial cytotoxic potential and are frequently enriched in immune-active TME (8). Recent studies across various solid tumors including colorectal, lung, esophageal, and head and neck cancers have demonstrated that the presence of Ttex, particularly when quantified relative to total CD8^+^ T cells (Ttex/CD8^+^ ratio), can serve as a prognostic indicator of patient outcomes (8, 9). These findings suggest that Ttex are not simply bystanders of immune dysfunction but may serve as valuable biomarkers of immune engagement and tumor control.

The emergence of multiplex imaging, spatial transcriptomics, and high-resolution single-cell profiling has enabled deeper interrogation of the localization, phenotype, and functional state of Ttex within the tumor milieu (10). Concurrently, digital pathology and machine learning tools offer promising avenues to quantify Ttex in a clinically applicable manner, bridging the gap between immunologic insights and diagnostic implementation (11, 12). These technological advances make this an opportune moment to consolidate current knowledge on Ttex, as their integration into biomarker-driven oncology and therapeutic design is now more feasible than ever.

In this review, we summarize the current understanding of terminally exhausted CD8^+^ T cells across solid tumors, with a focus on their prognostic relevance, immunogenomic correlates, and translational potential. We discuss how the Ttex/CD8^+^ ratio could be standardized into pathology workflows, explore strategies to target or modulate Ttex, and highlight future directions for integrating Ttex assessment into precision oncology.

## 2 Biology and definition of Ttex

Although exhaustion can occur in CD4^+^ T cells, γδ T cells, and NK-like T cells (11, 13), this review focuses specifically on CD8^+^ T cells because terminally exhausted CD8^+^ populations (Ttex) represent the most extensively characterized exhaustion state with the strongest biological, clinical, and translational relevance. T cell exhaustion represents a dynamic and heterogeneous state of CD8^+^ T cell dysfunction that arises in the context of chronic antigen exposure, such as during persistent viral infections or within the TME. Among the exhausted T cell subpopulations, Ttex are defined by distinct phenotypic, functional, and spatial characteristics that distinguish them from their progenitor exhausted (Tpex) counterparts (14). Increasing evidence demonstrates that exhaustion is not simply a terminal dysfunction, but a coordinated transcriptional, metabolic, and epigenetic program, shaped by persistent antigen, stromal cues, cytokine gradients, and tumor-derived metabolites (3, 15, 16). Ttex represent the fixed endpoint of this continuum, characterized by irreversible chromatin remodeling and profound metabolic impairment (17, 18).

### 2.1 Phenotypic markers

Ttex cells are typically characterized by sustained high expression of inhibitory receptors, including PD-1, TIM-3, and lymphocyte-activation gene 3 (LAG-3), coupled with the loss of the transcription factor TCF1 (encoded by Tcf7) (18–20). They also express exhaustion-associated transcriptional regulators such as thymocyte selection-associated high mobility group box protein (TOX), which are essential for establishing and stabilizing the exhausted state (21, 22). The PD1^high^TCF1^–^ phenotype has been widely used as a practical immunohistochemical signature for identifying Ttex in human tumors (23, 24).

A practical molecular distinction is that Tpex retain TCF1 (TCF1^+^ PD-1^int^ CXCR5^+^ SLAMF6^+^, TOX^low^), whereas Ttex lose TCF1 and upregulate co-inhibitory receptors such as TIM-3, LAG-3, and CD39, together with high TOX levels (25). Compared to Tpex cells, which retain self-renewal and proliferative potential, Ttex cells represent a more differentiated and fixed state of exhaustion.

Recent studies demonstrate that Ttex exhibit *de novo* accessible chromatin regions enriched for TOX and NR4A motifs, establishing an epigenetically locked exhaustion module (15, 26). In parallel, upregulation of CD39, CD103, and CXCL13 identifies Ttex clusters that preferentially localize to CAF-rich or metabolically stressed niches (27, 28). High-dimensional profiling also reveals multiple Ttex subsets (e.g., TOX^high^, ZNF683^high^, and CD38^hghi^ populations), highlighting that exhaustion encompasses multiple terminal states rather than a single uniform phenotype (29, 30).

### 2.2 Functional state

Despite their impaired cytokine production and proliferative capacity, Ttex cells are not entirely inert. They often retain residual cytolytic function, including expression of perforin and granzymes, and can contribute to tumor control under certain conditions (31). However, they are generally considered refractory to checkpoint blockade therapies, likely due to their epigenetically fixed exhaustion state (32, 33).

This functional limitation underscores the importance of maintaining a pool of Tpex cells for effective immune reinvigoration. Although Tpex share some features of exhaustion, they retain proliferative capacity, self-renewal, and lineage plasticity, acting as the progenitor population that continually replenishes the Ttex compartment, and responses to checkpoint blockade (34).

Recent mechanistic studies further show that Ttex undergo mitochondrial dysfunction driven by PGC1α suppression, impaired oxidative phosphorylation, and ROS accumulation (35, 36). Lipid peroxidation and cholesterol overload common features of the TME promote ferroptosis sensitivity in Ttex (37, 38). Additionally, chronic exposure to IL-10, TGF-β, and type I interferons reinforces the dysfunctional state by suppressing STAT5 and NF-κB signaling (39, 40). Together, these metabolic and cytokine-mediated constraints explain why Ttex rarely regain effector functions after PD-1 blockade.

### 2.3 Spatial context

Spatial localization profoundly shapes Ttex behavior. In multiple solid tumor types, Ttex cells accumulate within tumor nests or hypoxic stromal zones enriched with dysfunctional APCs and CAF-derived suppressive cues (3, 41, 42). In contrast, Tpex cells localize to peripheral regions, or tertiary lymphoid structures (TLS), where they remain proliferative and maintain therapeutic responsiveness (43, 44).

**Figure 1:**
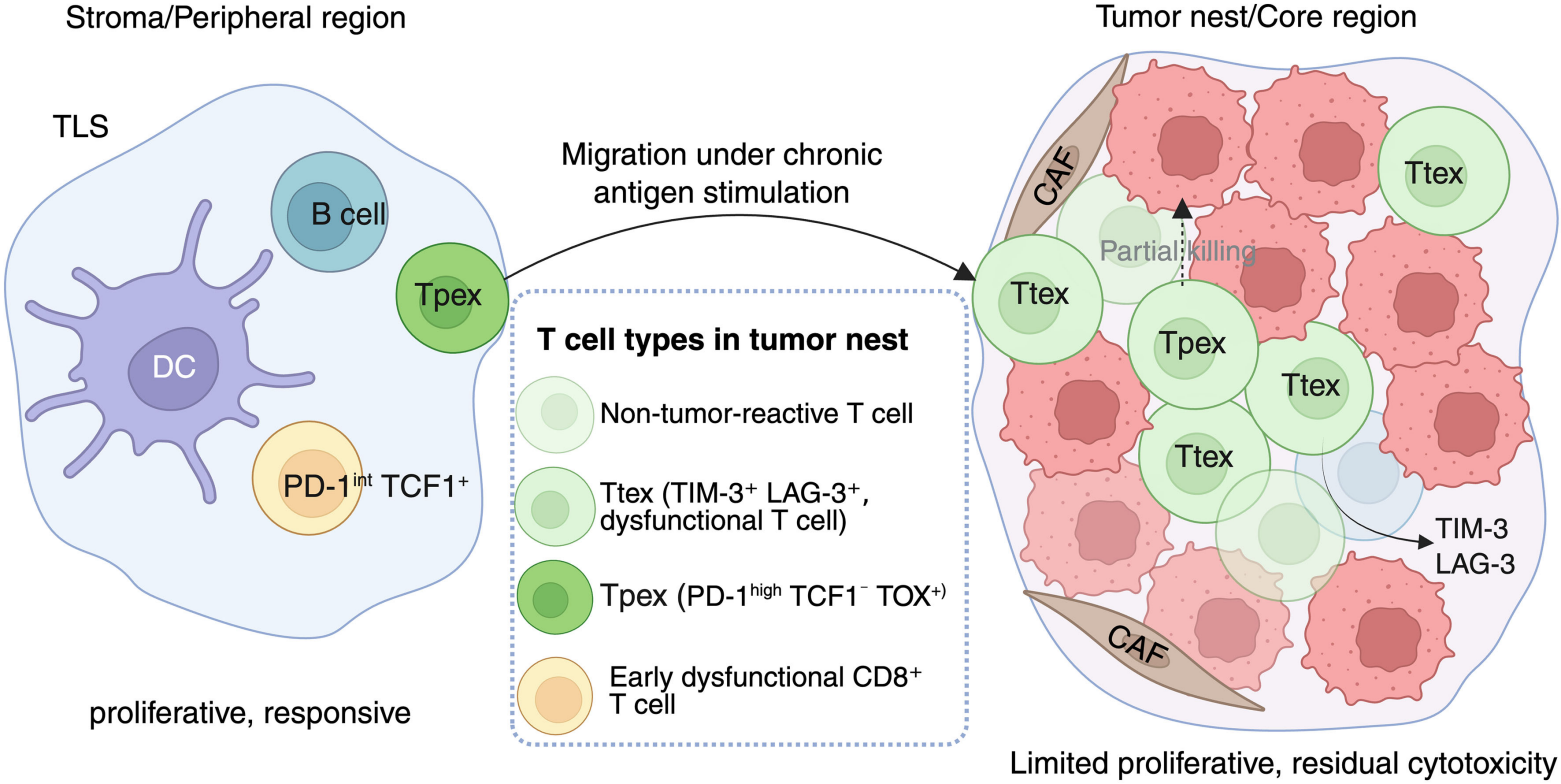
Spatial distribution of progenitor-like versus terminally exhausted CD8^+^ T cells in solid tumors. Schematic illustration depicting the organization of CD8^+^ T cell subsets across the TME. Left: Tpex cells reside predominantly in stromal or tertiary lymphoid structure (TLS) regions near the tumor boundary, where APC-rich niches support their proliferative capacity and maintain stem-like transcriptional programs (TCF1^+^PD-1^int^). Right: Ttex accumulate within the tumor nest/core region, often interspersed with non-tumor-reactive bystander T cells in CAF-rich and metabolically stressed niches. These Ttex cells (PD-1^high^, TCF1^–^, TOX^+^) exhibit limited proliferative capacity and residual cytotoxicity, reflecting advanced exhaustion and sustained immunosuppression. This spatial segregation highlights the distinct roles of Tpex and Ttex in immune surveillance, persistence, and therapeutic responsiveness.

Spatial-omics technologies show that Ttex preferentially reside in CAF-dominated niches enriched in CXCL12, TGF-β, and dense collagen, which restrict T cell movement and exclude Tpex (45). High-resolution imaging demonstrates that Ttex form tightly packed clusters adjacent to hypoxic pockets and metabolically stressed APCs, suggesting a stromal–metabolic ecosystem that reinforces exhaustion fixation (17). **Figure 1** provides a schematic overview of the spatial organization of progenitor exhausted (Tpex) and terminally exhausted (Ttex) CD8⁺ T-cell subsets within the tumor microenvironment, highlighting their distinct localization patterns and functional niches.

### 2.4 Differentiation pathway

The developmental trajectory of exhausted CD8^+^ T cells typically follow a stepwise progression from naive cells to effector cells, then to Tpex cells, and ultimately to Ttex cells (5, 8, 46). Tpex cells function as a self-renewing progenitor pool capable of generating Ttex progeny under chronic antigenic stimulation (47, 48). This linear differentiation is regulated by transcriptional programs, metabolic regulation, and microenvironmental cues, whin include cytokine availability (e.g., IL-21 and IL-10), antigen burden, and spatial immune architecture (28, 49).

**Figure 2:**
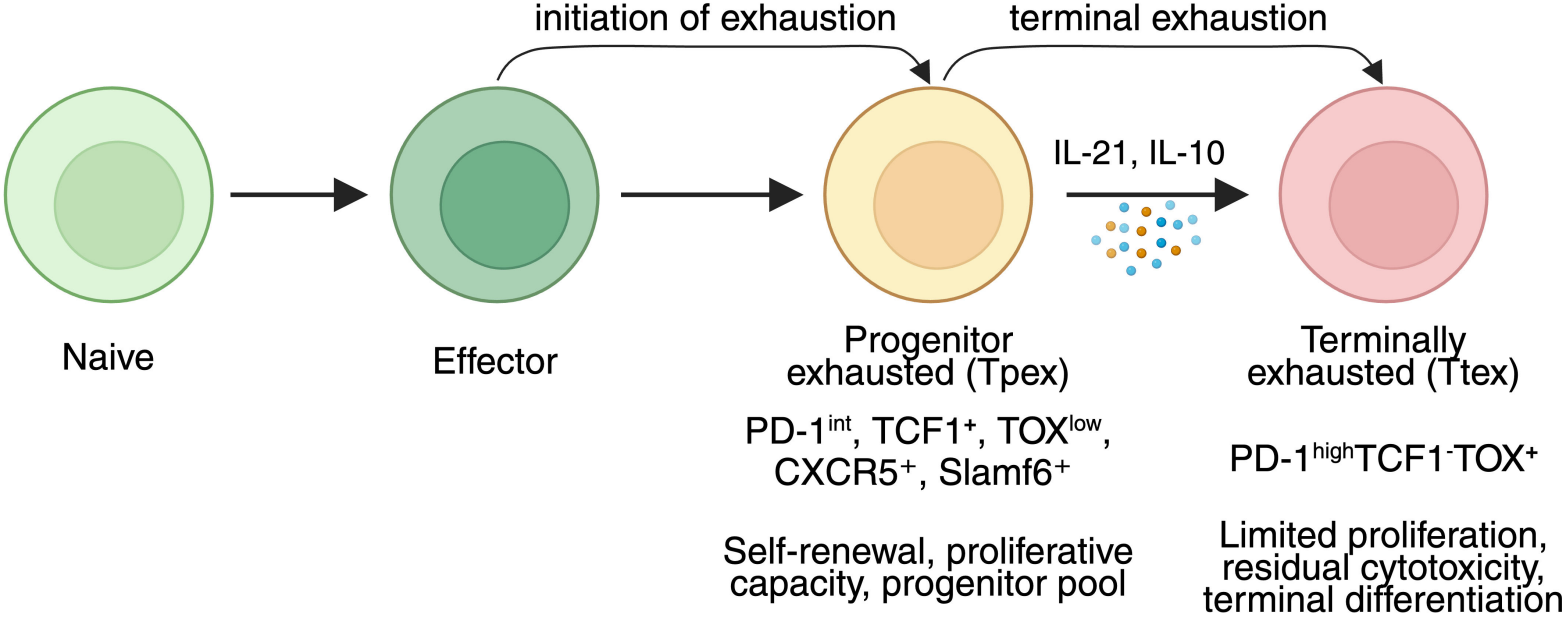
Stepwise differentiation of CD8^+^ T cells toward exhaustion under chronic antigen exposure. Naïve CD8^+^ T cells differentiate into effector cells, which upon chronic antigen stimulation progress into Tpex and Ttex states. Tpex cells (PD-1^int^, TCF1^+^, TOX^low^, CXCR5^+^, Slamf6^+^) maintain proliferative and self-renewal capacity and serve as a progenitor pool. With persistent antigenic stimulation and microenvironmental cues such as IL-21 and IL-10, Tpex cells give rise to Ttex cells (PD-1^high^, TCF1^–^, TOX^+^), which display limited proliferation and residual cytotoxicity. This trajectory highlights the balance between maintaining functional progenitor pools and terminal differentiation under chronic immune pressure.

A deeper understanding of the transition from Tpex to Ttex is essential for developing strategies that preserve T cell functionality and prevent irreversible exhaustion. Recent mechanistic advances show that Tpex fate is stabilized by TCF1, BCL6, and memory-associated chromatin accessibility, which collectively maintain proliferative potential and lineage plasticity (15, 26). In contrast, commitment to the Ttex lineage is driven by sustained NFAT signaling, TOX-mediated chromatin remodeling, and the loss of CCR7/CXCR5-guided migratory programs (17, 27), marking the shift from a progenitor-like to a tissue-resident terminal state. Meanwhile, microenvironmental pressures including AMPK–mTOR imbalance, mitochondrial depolarization, and lactate-rich acidification further bias Tpex cells toward terminal differentiation (35, 39). Together, these findings establish exhaustion as a coordinated fate decision shaped jointly by chronic antigen burden and stromal-metabolic cues (28). **Figure 2** illustrates the stepwise differentiation trajectory of CD8⁺ T cells under chronic antigen stimulation, from naïve and effector states through progenitor exhaustion (Tpex) to terminal exhaustion (Ttex).

## 3 Ttex across solid tumors: landscape and diversity

Ttex cells have been increasingly recognized as a distinct and functionally significant T cell population across a wide range of solid tumors. Advances in high-dimensional profiling technologies, including single-cell RNA sequencing (scRNA-seq), spatial transcriptomics, and multiplex immunohistochemistry (IHC), have enabled the detailed characterization of Ttex infiltration patterns, phenotypes, and prognostic implications in the TME (50, 51). While the presence of Ttex is consistently associated with chronic antigen stimulation, the extent, functional impact, and clinical significance of these cells vary across cancer types.

Across tumor types, Ttex commonly accumulate in chronically inflamed or antigen-rich regions, display conserved exhaustion signatures (TOX, PD-1^high^, TIM-3), and frequently localize within stromal or immune-excluded niches (18). However, their prognostic meaning diverges: MSI-H CRC and melanoma often show favorable associations linked to ongoing immune pressure, whereas NSCLC, HNSCC, ESCC, and HCC frequently demonstrate detrimental outcomes driven by stromal exclusion, CAF activity, or restricted Tpex reservoirs (8, 52). This review emphasizes tumor types with the most comprehensive single-cell or spatial data on Ttex (e.g., CRC, NSCLC, HNSCC, ESCC, melanoma, HCC). Breast and ovarian cancers are included briefly to illustrate the current knowledge gaps in less immune-inflamed tumors.

### 3.1 Colorectal cancer

In CRC, the biology and prognostic implications of Ttex differ markedly between microsatellite instability-high (MSI-H) and microsatellite stable (MSS) disease. In MSI-H CRC, which is characterized by high tumor mutational burden (TMB), and abundant neoantigens, Ttex infiltration is typically enriched and often colocalized with neoantigen-rich tumor regions. These Ttex cells often reside adjacent to tertiary lymphoid structures (TLS), and a higher Ttex/CD8^+^ ratio correlates with improved relapse-free survival, reflecting ongoing immune pressure despite features of exhaustion (9, 28). These findings demonstrates that the presence of exhausted cells does not necessarily signify immune failure but can instead mark active tumor–immune engagement.

In contrast, MSS CRC, which represents 85-90% of all CRC cases shows a different spatial and immunologic landscape (53, 54). MSS tumors generally have lower overall CD8^+^ T-cell infiltration and a reduced Ttex/CD8^+^ ratio compared with MSI-H CRC (55). Spatial profiling studies indicate that Ttex in MSS CRC are frequently confined to stromal or immune-excluded niches, with limited association with TLS or antigen-rich tumor nests (53, 56). These features reflect a weaker immune-reactive microenvironment and reduced availability of Tpex precursors capable of sustaining productive anti-tumor responses. As a result, Ttex abundance in MSS CRC often carries neutral or unfavorable prognostic implications, driven by stromal barriers, low neoantigen load, and restricted T cell access to tumor cores (8).

### 3.2 Non-small cell lung cancer

NSCLC is characterized by high mutational burden and frequent neoantigen presentation, providing fertile ground for T cell engagement and exhaustion (57, 58). Ttex cells in NSCLC exhibit PD-1^high^, TCF1^–^ phenotypes and often coexpress T-cell immunoglobulin and mucin domain-containing protein 3 (TIM-3) and other exhaustion markers (18, 59, 60). Their abundance has been associated with mixed outcomes: in some cohorts, high Ttex frequencies correlate with resistance to PD-1 blockade, while in others, their presence within TLS-rich regions suggests a role in maintaining long-term immune pressure (9, 23).

### 3.3 Head and neck squamous cell carcinoma

In HNSCC, Ttex cells have been identified as key components of immune-infiltrated tumors (3, 61). The presence of TCF1^–^PD-1^high^ CD8^+^ T cells in intraepithelial regions correlates with a suppressive immune landscape and poorer overall survival (62, 63). These findings suggest that in certain contexts, Ttex may serve as markers of immune evasion or progression, particularly when uncoupled from a supportive Tpex population or TLS architecture.

### 3.4 Esophageal squamous cell carcinoma

Recent spatial atlases of ESCC have revealed that Ttex cells emerge during high-grade intraepithelial neoplasia, coinciding with early signs of immune suppression and stromal remodeling (9, 64). These cells accumulate in CAF-enriched niches and contribute to the formation of immune-excluded microenvironments (65, 66). While their prognostic role remains under investigation, their early presence suggests a potential role in initiating immune evasion mechanisms (8, 16, 67).

### 3.5 Melanoma

Melanoma has been a prototypical model for T cell exhaustion studies due to its immunogenicity and responsiveness to checkpoint inhibitors (68, 69). Ttex cells are abundant in chronic lesions and often display a fixed epigenetic program that limits reinvigoration by PD-1 blockade (70). Nonetheless, their presence especially when balanced with a Tpex reservoir may be indicative of ongoing immune responses and partial tumor control (14, 69).

### 3.6 Hepatocellular carcinoma

HCC is characterized by a tolerogenic immune environment that favors T cell dysfunction. Ttex cells in HCC express exhaustion markers including TOX, CD39, and PD-1, and are enriched in the peritumoral stroma (71, 72). High densities of these cells have been linked to poor prognosis, particularly in the absence of Tpex or effector cell support (73, 74). These findings underscore the importance of cellular context in interpreting Ttex significance.

### 3.7 Breast and ovarian cancers

In breast and ovarian cancers, where immune exclusion is common, Ttex cells are often confined to stromal zones and may represent a frustrated immune attempt to access tumor nests (3, 75). Limited studies suggest that increased Ttex presence correlates with better prognosis, particularly in triple-negative breast cancer and BRCA1-mutated tumors, where T cell infiltration tends to be higher (76–78). However, more data are needed to clarify their functional role in these tumor types.

### 3.8 Prognostic variability across tumor types

The prognostic impact of Ttex varies by tumor type and immune context. In immune-inflamed tumors with high TMB or TLS presence, Ttex may reflect an active, ongoing immune response, correlating with favorable outcomes (8, 27, 79). Conversely, in immune-excluded or stromal-rich tumors, a predominance of Ttex may indicate T cell dysfunction and failure to control tumor growth, thereby associating with worse prognosis (3, 80, 81). This duality underscores the importance of interpreting Ttex in the context of stromal composition, spatial exclusion, and Tpex reservoir availability.

Across cancers, terminal exhaustion arises through shared mechanisms including TOX-driven chromatin remodeling, PD-1/TIM-3/LAG-3 co-expression networks, and depletion of stem-like Tpex precursors under chronic antigen exposure (26, 34). These conserved features define a unifying exhaustion axis that could underpin pan-cancer biomarker development.

**Figure 3:**
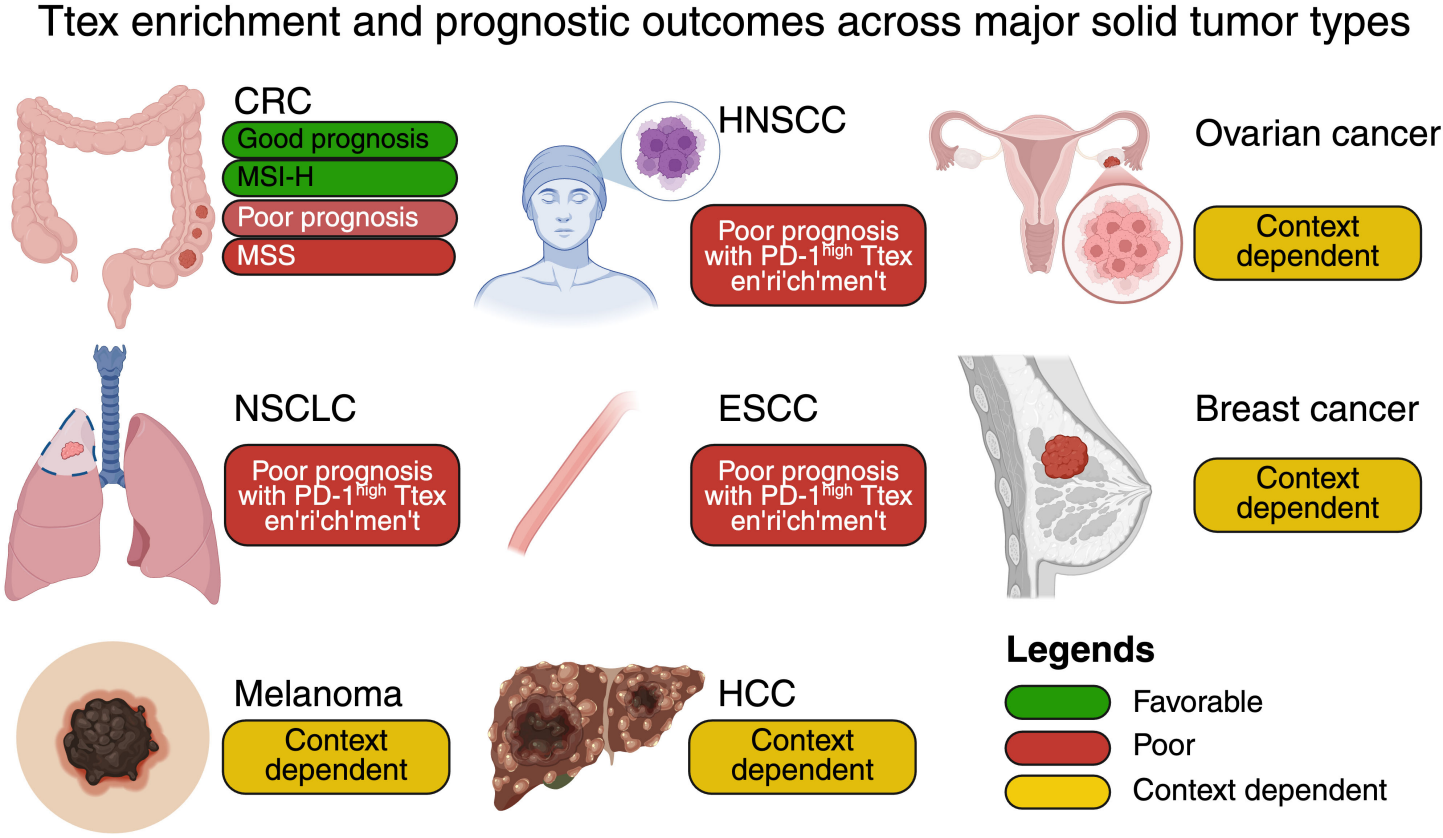
Ttex enrichment and prognostic outcomes across major solid tumors. Comparative schematic summarizing Ttex enrichment and prognostic outcomes across major solid tumor types. Favorable outcomes (green) are exemplified by MSI-H CRC, where TLS-associated Ttex support durable anti-tumor immunity. Poor prognosis (red) is linked to high accumulation of PD-1^high^ Ttex in non-small cell lung cancer (NSCLC), head and neck squamous cell carcinoma (HNSCC), and esophageal squamous cell carcinoma (ESCC), reflecting advanced exhaustion states. Mixed or context-dependent associations (yellow) are observed in melanoma, hepatocellular carcinoma (HCC), breast cancer, and ovarian cancer, where spatial distribution and immune contexture influence clinical outcomes.

At the same time, prognostic heterogeneity reflects variation in TLS density, stromal permissiveness, and availability of Tpex capable of differentiation (82–84). In immune-excluded or CAF-dominated niches, Ttex enrichment more often marks immune failure rather than immune pressure (85, 86). These distinctions highlight the need to contextualize Ttex biology within both spatial and immunogenomic frameworks. **Figure 3** summarizes the enrichment of Ttex cells and their tumor-context-dependent prognostic associations across major solid tumor types.

## 4 Immunogenomic and spatial correlates

The presence, phenotype, and prognostic significance of Ttex in solid tumors are closely intertwined with the immunogenomic landscape and spatial architecture of the TME. Advances in multi-omics and spatial profiling technologies have revealed that Ttex abundance is not random but rather aligned with specific tumor-intrinsic features and localized immune structures (87–89). Understanding these associations provides a foundation for integrating Ttex-based metrics into biomarker strategies for precision oncology.

### 4.1 Association with MSI

MSI-H tumors, particularly in colorectal and endometrial cancers, are characterized by elevated mutation rates and robust immune infiltration (90, 91). These tumors often exhibit increased frequencies of Ttex, likely driven by chronic stimulation from a high neoantigen burden (92, 93). Notably, in MSI-H CRC, a higher Ttex/CD8^+^ ratio has been associated with improved relapse-free survival, suggesting that the presence of Ttex reflects a sustained immune response rather than immune failure (4, 6, 8). This observation contrasts with the traditional assumption that T cell exhaustion equates to dysfunction, highlighting the importance of genomic context in interpreting exhaustion phenotypes.

Recent MSI-focused spatial transcriptomics studies show that frameshift neoantigens induce chronic TCR signaling, driving TOX and NR4A expression and promoting Tpex to Ttex differentiation (94). MSI tumors also exhibit distinct cytokine gradients, particularly IL-21 and type I IFNs that sustain progenitor pools yet simultaneously promote terminal exhaustion in high-antigen niches (95, 96).

### 4.2 TMB and neoantigen load

High TMB has been linked to increased T cell infiltration, including the recruitment and differentiation of CD8^+^ T cells into an exhausted state (1, 97). Ttex are frequently enriched in TMB-high tumors, where continuous antigen exposure drives terminal differentiation (3, 23). Similarly, high neoantigen load has been positively correlated with Ttex abundance across several solid tumor types, including melanoma, NSCLC, and MSI-H CRC (3, 8, 98). These associations suggest that Ttex presence may serve as a surrogate marker for immunogenicity, particularly in settings where direct neoantigen profiling is not feasible.

High TMB tumors frequently harbor clonal neoantigens that sustain NFAT-dependent calcium signaling, a key driver of TOX upregulation (27, 57). Single-cell analyses further show that TMB-high tumors enrich for CXCL13^+^ Ttex clusters associated with exhausted yet tumor-reactive T cell clones (99, 100).

### 4.3 Tertiary lymphoid structures

Ttex often exist in juxtaposition to TLS organized lymphoid aggregates that serve as local hubs for antigen presentation and T cell priming (43). In tumors with mature TLS, Tpex cells are thought to originate or persist within these niches, while Ttex localize more distally within tumor nests, reflecting their terminal differentiation status (14, 83). The spatial relationship between TLS and Ttex may have prognostic implications (43, 44, 101). Tumors with coexisting TLS and Ttex infiltration often show improved clinical outcomes, suggesting a dynamic interplay between T cell priming and effector exhaustion within the tumor ecosystem (28, 43).

Recent deep-imaging studies reveal that TLS-associated dendritic cells deliver IL-21 and CD28 co-stimulation that preserve Tpex differentiation (102). Loss of TLS or spatial decoupling from antigen-presenting cells accelerates Tpex exhaustion and Ttex accumulation, altering clinical outcomes (14, 103).

### 4.4 High-resolution mapping technologies

The integration of high-dimensional spatial and single-cell tools has revolutionized the characterization of Ttex within the TME (86, 104). Multiplex immunofluorescence (mIF) enables simultaneous detection of multiple exhaustion and activation markers, such as PD-1, TCF1, TOX, and Granzyme B, within formalin-fixed tissues, thereby providing both quantitative and spatial information on Ttex in clinical samples (105). Spatial transcriptomics extends this capability by preserving tissue architecture while generating gene expression maps, allowing exhaustion gene signatures including TOX, LAG3, and CD39 to be localized to tumor margins, immune niches, or stromal barriers (106). scRNA-seq combined with protein barcoding technologies such as Cellular Indexing of Transcriptomes and Epitopes by sequencing (CITE-seq) or Cytometry by time-of-flight (CyTOF) offers even deeper resolution, capturing the transcriptional continuum from naïve to Tpex and Ttex states, as well as clonal relationships and functional potential (107).

Together, these approaches provide a nuanced understanding of how tumor-intrinsic features, such as MSI and TMB, interact with spatial immune architecture, including TLS, to shape the formation and persistence of Ttex. Collectively, they establish the foundation for biomarker development and patient stratification strategies based on T cell exhaustion states. A summary of these technologies, including their strengths, limitations, and clinical applicability, is provided in **Table 1**.

**Table 1:** Technologies for detecting and quantifying Ttex cells.

Technology	Key markers	Strengths	Limitations	Clinical applicability	References
Multiplex immune fluorescence (mIF)	PD-1, TCF1, TOX, TIM-3, Granzyme B	Allows simultaneous detection of multiple markers in FFPE tissues; preserves spatial context	Requires specialized equipment and expertise; interpretation may vary across labs	Increasingly adopted in translational studies; compatible with clinical pathology	(108, 109)
Multiplex immune-histochemistry (mIHC)	PD-1, TCF1, CD8, TOX	Cost-effective and compatible with routine pathology workflows; suitable for FFPE samples	Lower multiplexing capacity compared to mIF; potential antibody cross-reactivity	High; can be integrated into diagnostic pathology with AI-enabled quantification	(110, 111)
Spatial transcriptomics	Exhaustion signatures (TOX, LAG3, CD39, TIGIT)	Preserves tissue architecture while mapping gene expression; enables TME-wide profiling	High cost, limited resolution (~10–50 μm); requires fresh/frozen samples	Currently research-oriented; clinical adoption pending validation	(43, 112)
scRNA-seq with protein barcoding (CITE-seq/CyTOF)	PDCD1, TCF7, TOX, GZMB + surface proteins	High-resolution profiling of T cell states and trajectories; links transcriptome with clonal information	Expensive, labor-intensive; requires fresh tissue; limited scalability	Research only; provides mechanistic insights for biomarker discovery	(11, 113)
Digital pathology with AI analysis	Composite panels (PD-1^high^, TCF1^−^, TOX, CD39)	Automated, reproducible quantification; scalable for multicenter validation	Dependent on training datasets and algorithm quality; requires digital infrastructure	Promising for clinical biomarker development; bridges research and pathology	(114, 115)

## 5 Prognostic value of Ttex and the Ttex/CD8^+^ ratio

The prognostic significance of Ttex cells has gained increasing attention in recent years, challenging traditional assumptions that exhaustion necessarily equates to immune failure (116). Instead, accumulating evidence suggests that the presence of Ttex especially when contextualized relative to total CD8^+^ T cell infiltration can serve as a meaningful biomarker of immune engagement and tumor control in multiple solid tumor types (28, 117).

### 5.1 Clinical outcomes associated with Ttex presence

Several retrospective and prospective studies across different tumor types have demonstrated that Ttex infiltration is associated with favorable clinical outcomes under certain conditions (118, 119). In MSI-H CRC, for example, the enrichment of PD-1^high^ TCF1^−^ CD8^+^ T cells within the tumor epithelium correlates with prolonged relapse-free survival (RFS) (120). Similarly, in NSCLC, the presence of Ttex has been linked to sustained immune pressure and improved survival in subsets of patients with immune-infiltrated tumors (6, 121). These findings underscore the importance of interpreting Ttex not in isolation but within the broader immune context.

### 5.2 Ttex/CD8^+^ ratio as a prognostic biomarker

Recent suggests that the Ttex/CD8^+^ ratio, rather than absolute number of Ttex cells, provides a more accurate reflection of the immune landscape within solid tumor (9, 69, 122). A high ratio indicates that a substantial proportion of infiltrating CD8^+^ T cells have progressed to terminal exhaustion, often reflecting chronic antigen exposure and prior immune activation (1, 9, 123). Importantly, the prognostic significance of this ratio is strongly cancer-type dependent. In MSI-H CRC, a higher Ttex/CD8^+^ ratio consistently predicts favorable outcomes and reduced recurrence risk, even when total CD8^+^ infiltration is modest (28, 124). In contrast, in NSCLC, HNSCC, and ESCC, elevated Ttex/CD8^+^ ratios are generally associated with poor prognosis, reflecting stromal exclusion, insufficient Tpex support, and dysfunctional immune pressure (61, 113, 125). Melanoma shows a more context-dependent pattern, where Ttex enrichment can be favorable when accompanied by robust Tpex reservoirs but unfavorable in immunosuppressed or immune-excluded tumors (34, 126). In hepatocellular carcinoma, high Ttex/CD8^+^ ratios correlate with reduced survival, consistent with the tolerogenic hepatic microenvironment (127, 128). Conversely, in triple-negative and BRCA1-mutant breast cancers, higher Ttex/CD8^+^ ratios may indicate active anti-tumor immunity in highly infiltrated tumors (129, 130). Collectively, these findings demonstrate that the Ttex/CD8^+^ ratio is a tumor-context-specific biomarker whose prognostic interpretation depends on mutational burden, stromal architecture, and the presence of supportive progenitor Tpex pools (131). Importantly, the same Ttex/CD8^+^ ratio may reflect fundamentally different biological states across tumor types, ranging from ongoing immune engagement to terminal immune failure, underscoring the necessity of tumor-context-specific interpretation.

### 5.3 Thresholds and risk stratification

While the Ttex/CD8^+^ ratio shows considerable promise as a prognostic biomarker, standardized thresholds for clinical use have not yet been defined (132, 133). Most existing studies rely on cohort-specific medians or quartiles to classify patients into high- versus low-ratio groups, which limits comparability across cohorts (134). To enable broader adoption, future efforts should establish universal cutoffs validated across diverse datasets and platforms. In addition, developing automated scoring systems using digital pathology or AI-assisted quantification, and integrating the ratio with contextual features such as proximity to tumor cells or the presence of tertiary lymphoid structures, will further enhance its clinical utility (135, 136). Together, these advances could support the incorporation of Ttex-based metrics into routine risk stratification models, particularly for patients undergoing adjuvant therapy or clinical surveillance. A summary of the prognostic associations of Ttex across solid tumors is provided in **Table 2**.

**Table 2:** Prognostic significance of Ttex cells across solid tumors.

Tumor type	Ttex markers	Spatial localization	Prognostic association	Notes	References
CRC, especially MSI-H	PD-1^high^, TCF1^−^, TOX	Neoantigen-rich tumor regions, intraepithelial niches	Higher Ttex/CD8^+^ ratio linked with improved relapse-free survival	Reflects active immune surveillance despite low total CD8^+^ density	(137, 138)
NSCLC	PD-1^high^, TCF1^−^, TIM-3	TLS-rich regions or intratumoral cores	Mixed: favorable when associated with TLS; resistance to PD-1 blockade in some cohorts	Context-dependent; Tpex reservoir critical for benefit	(139, 140)
HNSCC	PD-1^high^, TCF1^−^	Intraepithelial tumor regions	Poor prognosis with high intraepithelial Ttex	Reflects suppressive immune landscape lacking Tpex support	(141, 142)
ESCC	PD-1^high^, TCF1^−^, TOX	CAF-enriched, immune-excluded niches	Associated with immune evasion and poor survival	Detected in early neoplastic stages, may signal onset of immune exclusion	(143, 144)
Melanoma	PD-1^high^, TCF1^−^, TOX	Chronic lesions, tumor nests	Abundant Ttex often refractory to PD-1 blockade; partial tumor control possible	Prognostic role depends on balance with Tpex reservoir	(24, 145)
HCC	PD-1, TOX, CD39	Peritumoral stroma	Poor prognosis, especially without Tpex/effector support	Tolerogenic microenvironment favors Ttex predominance	(146, 147)
Breast cancer (esp. TNBC)	PD-1, TOX, TCF1^−^	Stromal zones, peri-tumoral regions	Increased Ttex may correlate with better prognosis in TNBC and BRCA1-mutant tumors	Limited studies; immune infiltration context-dependent	(148, 149)
Ovarian cancer	PD-1, TCF1^−^	Stromal, immune excluded niches	Suggestive of improved outcomes in subsets with higher infiltration	More data needed for validation	(150, 151)

### 5.4 Comparison with established immune biomarkers

Compared with widely used immune biomarkers such as immunoscore (based on CD3^+^ and CD8^+^ cell densities) and PD-L1 IHC, the Ttex/CD8^+^ ratio provides complementary and additional insight into the functional state of the CD8^+^ T cell compartment (152, 153). The immunoscore primarily quantifies T-cell abundance, while PD-L1 IHC reflects expression of a single inhibitory ligand on tumor or immune cells (154). In contrast, quantifying Ttex cell captures information about CD8^+^ T cell differentiation, exhaustion trajectory, and functional competence, providing a layer of biological interpretation not captured by quantity-based or single-marker assays (18, 69).

Importantly, the Ttex/CD8^+^ ratio is not intended to replace existing biomarkers. Instead, it may refine clinical patient stratification by adding resolution within intermediate-risk groups, including those patients with modest CD8^+^ infiltration or ambiguous PD-L1 status, thereby offering incremental prognostic and mechanistic value when used alongside established tools (117, 150).

## 6 Translational applications and integration into clinical practice

As the landscape of cancer immunotherapy continues to evolve, there is growing interest in translating immune biomarkers from research setting to real-world clinical practice (155, 156). Ttex cells and their relative abundance within the tumor immune context hold promise as prognostic and predictive tools, particularly when incorporated into modern pathology workflows (132, 157). Advances in digital pathology, artificial intelligence (AI), and multiplex imaging technologies now enable standardized quantification of Ttex in formalin-fixed paraffin-embedded (FFPE) tumor specimens (158, 159). However, successful translation also requires critical evaluation of technical, analytical, and regulatory barriers that may limit reproducibility and broader adoption.

### 6.1 Digital pathology and AI-enabled Ttex quantification

The identification of Ttex cells typically defined by PD-1^high^ and TCF1^–^ expression can be achieved using multiplex immunofluorescence (mIF) or multiplexed IHC (mIHC) panels (160, 161). When coupled with AI-based image analysis platforms, these techniques allow automated detection, phenotyping, and spatial localization of Ttex within tumor tissues (162). Recent studies have demonstrated the feasibility of digital quantification of exhausted T cell subsets across large clinical cohorts (163). Incorporating additional markers such as CD8, TOX, and TIM-3 further enhances assay robustness and enables reproducible measurement of the Ttex/CD8^+^ ratio (18, 164).

AI-assisted systems hold promise for standardized Ttex/CD8^+^ quantification, though validation across platforms remains an active area of investigation (165, 166). Technical limitations remain an important consideration in translating multiplex imaging and AI-based Ttex quantification into clinical use (167, 168). Significant inter-platform variability such as differences observed across Akoya, NanoString, and Leica systems can influence dynamic range, fluorophore stability, and antibody compatibility, ultimately affecting the reliable detection of exhaustion markers (167, 169). In addition, the absence of universally standardized antibody panels, particularly the inconsistent performance of commonly used clones for PD-1, TIM-3, and TOX, introduces site-specific bias that complicates cross-study comparison (60). AI-assisted pathology also faces notable challenges, including algorithmic model drift, limited generalizability across institutions with different staining or scanning protocols, and variability in training datasets that may compromise reproducibility (170). Furthermore, regulatory barriers remain substantial, as diagnostic-grade digital pathology and AI classifiers require formal CE-IVDR or FDA clearance before widespread deployment (171). Together, these limitations highlight the urgent need for assay harmonization, rigorous inter-laboratory calibration, and multicenter standardization frameworks before Ttex quantification can be reliably incorporated into routine clinical workflows.

**Figure 4:**
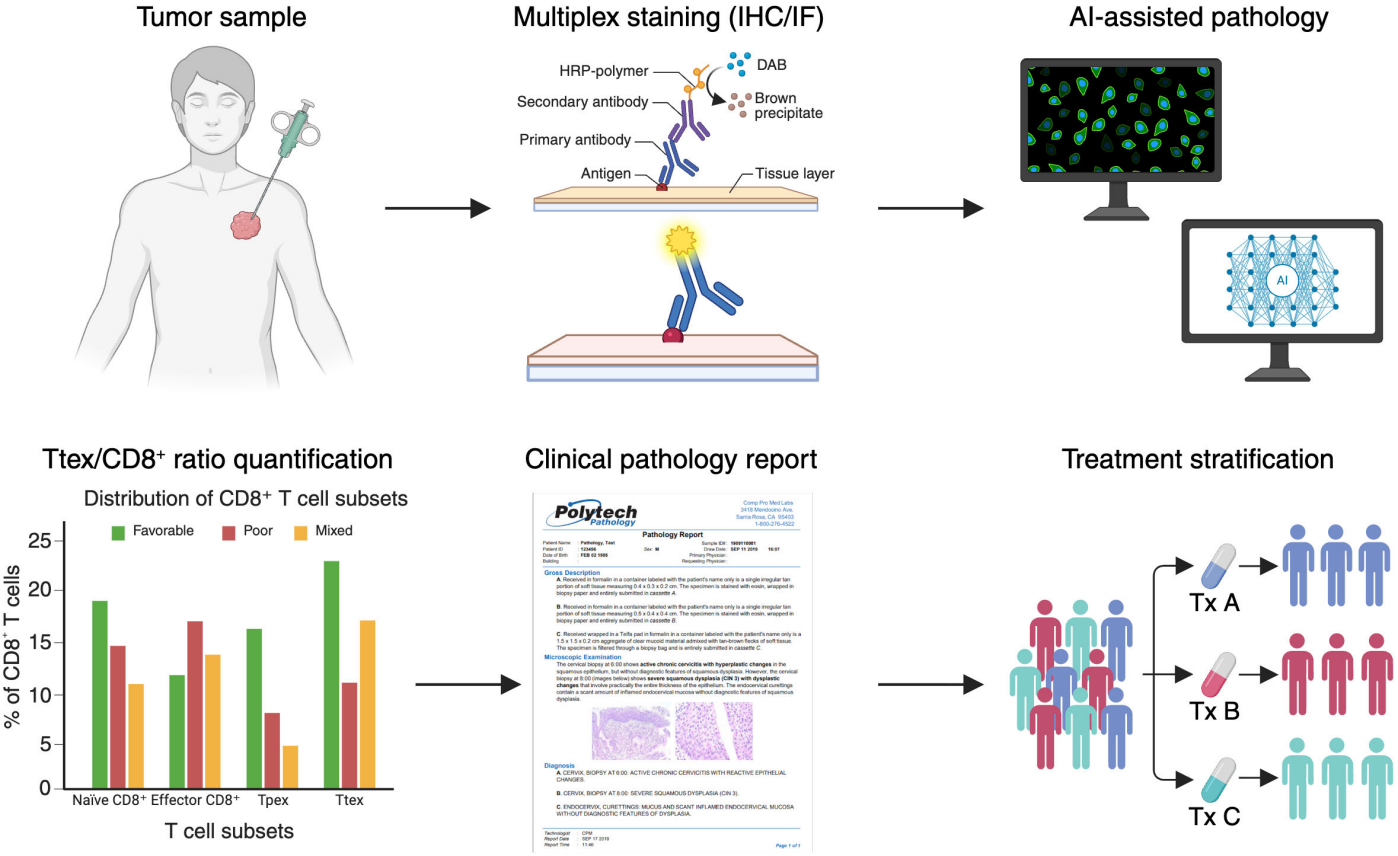
Workflow for Ttex quantification in clinical practice. Schematic illustration of a proposed translational pipeline integrating Ttex evaluation into clinical pathology. Tumor samples are processed using multiplex IHC or immunofluorescence (IF), followed by AI-assisted digital pathology for automated cell recognition. Quantification of the Ttex/CD8^+^ ratio is performed to distinguish between favorable, poor, and mixed prognostic groups. Results are incorporated into the clinical pathology report and used for treatment stratification, guiding decisions such as allocation to therapeutic regimens (Tx A, Tx B, Tx C).

To bridge these technical considerations with practical implementation, the following workflow summarizes how multiplex staining and AI-assisted pathology can be integrated to generate clinically actionable Ttex assessments. **Figure 4** outlines a representative workflow integrating multiplex staining, AI-assisted pathology, and quantitative analysis to derive clinically actionable Ttex/CD8⁺ metrics from patient tumor samples.

### 6.2 Feasibility in routine pathology workflows

The integration of Ttex scoring into diagnostic pipelines is increasingly feasible due to the widespread availability of whole-slide imaging, cloud-based computation, and digital biomarker platforms (86, 158). Unlike gene expression-based biomarkers, which often require RNA preservation or sequencing infrastructure, Ttex detection through protein-level multiplex staining is compatible with standard FFPE tissue and readily integrates into existing clinical workflows (172, 173).

However, several practical barriers must be acknowledged. Staining quality can vary across institutions due to differences in antigen-retrieval protocols and tissue-preservation methods, leading to inconsistent detection of exhaustion markers (11, 174). Batch effects introduced during multiplex staining or spectral unmixing further complicate cross-cohort comparability. Computational reproducibility also remains challenging, as analytical pipelines differ in segmentation models, thresholding strategies, and feature-extraction parameters (175). Furthermore, regulatory requirements add another layer of complexity, as AI-based diagnostic algorithms must undergo formal validation before clinical deployment (176, 177). Addressing these challenges through centralized AI models, ring-trial calibration, and consensus scoring guidelines will be essential for enabling large-scale and standardized clinical adoption.

### 6.3 Potential in adjuvant therapy decision-making

The prognostic value of Ttex, especially when evaluated using the Ttex/CD8^+^ ratio, may inform adjuvant therapy decisions in multiple tumor types (28, 178). In CRC, for example, high Ttex ratios have been associated with improved relapse-free survival, suggesting that these tumors may already be under active immunologic surveillance and may require less aggressive adjuvant treatment (179, 180). Conversely, tumors with low Ttex infiltration may benefit from escalated therapy or referral into clinical trials for immunomodulatory agents (181).

Similarly, in ESCC, spatial mapping studies demonstrate that early accumulation of Ttex coincide with immune exclusion (182, 183), identifying high-risk patients who may require closer monitoring or early intervention (43). Integration of Ttex profiling with existing clinicopathologic parameters may therefore enhance risk stratification beyond conventional staging systems (8, 27). At present, Ttex-based stratification strategies should be regarded as investigational, as prospective interventional trials validating their direct utility in clinical decision-making are still lacking.

### 6.4 Companion biomarker for immunotherapy and CAR-T cell trials

Beyond prognosis, Ttex metrics may serve as companion biomarkers for immune checkpoint inhibitors (ICIs) and chimeric antigen receptor T (CAR-T) therapies, especially in solid tumors where validated predictive biomarkers of response remain scarce (106, 184, 185). Ttex metrics are currently being explored as investigational biomarkers in ICI and CAR-T trials, with no regulatory approval to date. High Ttex abundance may indicate chronic antigen exposure and prior immune activation, identifying patients more likely to respond to PD-1/PD-L1 blockade or T cell reinvigoration strategies (186, 187). In contrast, a low Ttex/CD8^+^ ratio could signal insufficient T cell priming or immune exclusion, potentially guiding the selection of combination regimens such as anti-angiogenic agents, toll-like receptor (TLR) agonists, or stromal modulators (117, 188).

Additionally, in CAR-T cell trials, the pre-existing T cell exhaustion status in the TME may significantly influence CAR-T engraftment, expansion, and therapeutic durability (189, 190). Quantifying Ttex levels before treatment could enable more effective patient stratification and guide rational CAR design strategies aimed at resistant to exhaustion, such as modified co-stimulatory domains or exhaustion-resistant transcriptional programs (3, 191).

## 7 Therapeutic targeting and modulation of Ttex cells

Ttex cells are traditionally considered to be at the end of the exhaustion spectrum, with limited proliferative potential and responsiveness to immune checkpoint blockade (43, 192). However, recent research suggests that Ttex cells are not entirely inert and may still retain cytotoxic function or serve as indicators of past immune engagement (69, 74). This raises the important question: Can Ttex cells be reactivated or therapeutically modulated? (4, 193) While Tpex cells remain the primary responders to PD-1/PD-L1 inhibition, strategies aimed at enhancing or sustaining the functional capacity of Ttex cells are emerging and hold promise for improving immunotherapeutic outcomes (14, 194).

### 7.1 Can Ttex be reinvigorated?

Clinical and preclinical data suggest that Ttex are less responsive to checkpoint inhibitors than Tpex cells due to a fixed epigenetic state characterized by chromatin remodeling, TOX-driven transcriptional reprogramming, and metabolic exhaustion (195–197). Nonetheless, Ttex can still exert limited effector functions such as cytotoxicity and IFN-γ secretion, particularly in antigen-rich environments (198, 199). Recent studies show that PD-1 blockade primarily expands Tpex cells (200, 201), whereas Ttex, despite their limited proliferative capacity, can transiently support tumor control through short-lived cytotoxic activity (14, 187). Thus, Ttex cells may be therapeutically relevant in combination with approaches that prevent their accumulation, delay their terminal differentiation, or restore partial function (202). Thus, while Ttex retain limited residual effector capacity, current evidence does not support reliable functional reinvigoration of fully terminally exhausted T cells in patients, and most durable clinical responses appear to be driven by the Tpex compartment.

### 7.2 Emerging therapeutic strategies

Beyond conventional checkpoint blockade, several emerging strategies are being investigated to modulate or overcome terminal exhaustion. Combination checkpoint inhibitors targeting molecules frequently co-expressed with PD-1, such as TIM-3, LAG-3, and TIGIT, are showing promise in both preclinical and clinical studies, with the potential to address exhaustion at multiple stages (1, 203–205). Targeting transcriptional regulators, particularly TOX and the NR4A family, has demonstrated preclinical efficacy in delaying exhaustion and restoring partial effector function (206). Epigenetic reprogramming strategies, including siRNA or CRISPR-based modulation, also aim to reverse exhaustion-associated chromatin remodeling (207). In addition, non-coding RNAs such as circRNAs are emerging as novel regulators of T cell persistence and differentiation, representing potential tools for reprogramming exhaustion pathways (89). Engineering exhaustion-resistant T cells through CAR-T constructs with optimized costimulatory domains or CRISPR-mediated deletion of inhibitory genes offers another avenue to improve persistence and efficacy in solid tumors (208). Finally, metabolic modulation strategies targeting mitochondrial function or mTOR signaling may help sustain T cell fitness under chronic antigen stimulation (209).

**Figure 5:**
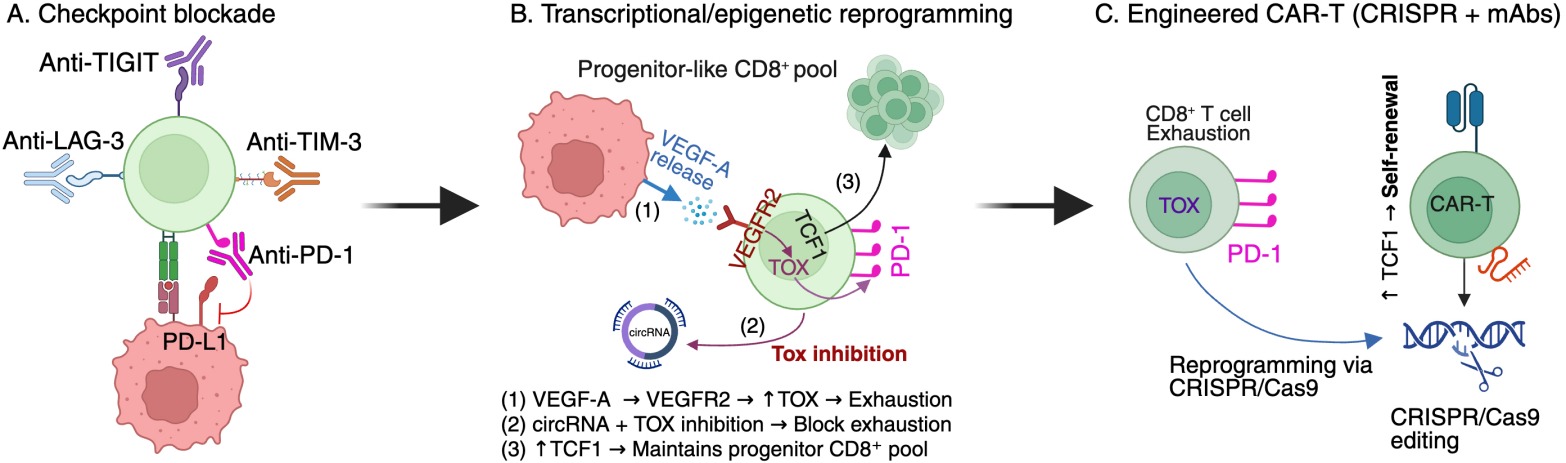
Therapeutic strategies targeting Ttex. Schematic overview of approaches to modulate Ttex in cancer immunotherapy. **(A)** Checkpoint blockade therapies targeting PD-1, TIM-3, TIGIT, or LAG-3 to alleviate inhibitory signaling. **(B)** Transcriptional and epigenetic reprogramming strategies aimed at preserving Tpex pools and limiting terminal exhaustion (15). Specifically (1), VEGFA-VEGFR2 signaling promotes TOX induction and exhaustion differentiation (210, 211) (2); circRNA- or small-molecule-mediated TOX inhibition suppresses exhaustion-associated transcriptional programs (212, 213); and (3) maintenance of TCF1 expression preserves progenitor exhausted CD8^+^ T cell pool and sustains long-term immune responsiveness (214, 215). **(C)** Engineered CAR-T cells, using CRISPR/Cas9-based genome editing and monoclonal antibody-based modulation to generate exhaustion-resistant T cells with enhanced self-renewal capacity and antitumor activity (216). Together, these complementary strategies aim to overcome terminal T cell exhaustion and improve the durability and efficacy of cancer immunotherapy.

To better conceptualize these approaches, **Figure 5** provides a schematic overview of therapeutic strategies aimed at overcoming terminal exhaustion, including checkpoint blockade, transcriptional/epigenetic reprogramming, and engineered CAR-T cells. This figure highlights how interventions can either block inhibitory signaling, reprogram exhaustion pathways, or engineer resistance to exhaustion, thereby enhancing T cell persistence and antitumor activity.

**Table 3** provides a concise summary of emerging therapeutic strategies, outlining their molecular targets, supporting preclinical and clinical evidence, and translational potential in cancer immunotherapy. This overview complements the schematic by serving as a quick reference for the scope and current maturity of each approach.

**Table 3:** Emerging therapeutic strategies targeting Ttex cells.

Strategy	Target(s)	Preclinical/clinical evidence	Potential applications	References
Combination checkpoint blockade	PD-1 + TIM-3, TIGIT, LAG-3	Ongoing clinical trials: preclinical synergy observed in NSCLC, melanoma	Overcoming late-stage exhaustion by blocking multiple inhibitory pathways	(217, 218)
Transcriptional modulation	TOX, NR4A family	Preclinical studies show TOX inhibition delays exhaustion; NR4A deletion improves T cell persistence	Enhancing effector function and delaying terminal exhaustion	(211, 219)
Epigenetic reprogramming	Chromatin accessibility (TOX-driven programs)	Experimental CRISPR/siRNA tools; reversible epigenetic remodeling reported	Restoring partial effector function in Ttex; improving responsiveness to ICIs	(220, 221)
Regulatory non-coding RNAs (circRNAs)	circRNAs regulating exhaustion-associated genes	circRNAs as T cell differentiation switches	Potential for transcriptomic reprogramming of endogenous T cells or enhancing CAR-T persistence	(89, 222)
Engineering exhaustion-resistant T cells	CAR-T constructs with 4-1BB, CRISPR edits of TOX/PD-1	Preclinical CAR-T models in solid tumors; clinical adoption in early-phase trials ongoing	Improving CAR-T engraftment, durability, and efficacy in solid tumors	(208, 223)
Metabolic modulation	mTOR, mitochondrial fitness pathways	Preclinical data in chronic infection and tumor models	Supporting T cell fitness and delaying exhaustion under chronic antigen exposure	(224, 225)

### 7.3 Toward rational combination therapies

Given the multifactorial nature of T cell exhaustion, it is increasingly clear that combination strategies are more likely to yield durable benefits than single-agent interventions (226). Rational pairings under investigation include PD-1 blockade with TIM-3 or TIGIT inhibition to simultaneously address early and late checkpoints of exhaustion, as well as combinations of checkpoint inhibitors with metabolic modulators that enhance mitochondrial fitness or regulate mTOR signaling (16, 60). Another promising direction involves integrating immunotherapies with circRNA-targeting agents to reprogram exhaustion pathways at the transcriptional level (227, 228). Importantly, Ttex profiling before and during treatment could serve as a guide for patient selection, ensuring that these combination strategies are applied to individuals most likely to benefit (50). Such biomarker-driven approaches represent a step toward precision immunotherapy, in which exhaustion status informs both therapeutic design and clinical decision-making.

## 8 Challenges and future directions

Despite growing interest in Ttex cells as prognostic and translational biomarkers in solid tumors, several key challenges limit their widespread clinical application. To fully realize the potential of Ttex-based strategies in precision oncology, both technical and conceptual gaps must be addressed through coordinated research and clinical efforts.

### 8.1 Inconsistent marker definitions across platforms

One of the foremost challenges is the lack of standardized phenotypic definitions for Ttex across different experimental and clinical platforms (6). While PD-1^high^ and TCF1^−^ expression is widely used as a practical definition in multiplex immunofluorescence and flow cytometry studies, these markers do not fully capture the diversity of terminally exhausted states (23). Additional markers such as TOX, CD39, TIM-3, and TIGIT are variably included, and their expression patterns fluctuate depending on tissue site, tissue type, disease stage, and staining platform (229, 230). Variability in antibody clones, thresholds, and gating strategies further complicates cross-study comparisons and limits the development of universal diagnostic assays (231). To overcome these inconsistencies, future work must prioritize the development of harmonized, multi-center antibody panels and standardized staining and scoring pipelines, enabling more reproducible identification of Ttex across institutions and imaging platforms.

### 8.2 Need for functional validation

Most studies to date have inferred Ttex identity and function based on marker expression rather than direct assessment of cytotoxic capacity or cytokine production. While exhausted T cells are often assumed to be dysfunctional, emerging evidence indicates that Ttex retain residual activity under certain conditions (193, 232). Functional assays, including single-cell cytokine profiling, degranulation (e.g., CD107a), and cytotoxicity assays, are needed to validate the effector potential of Ttex across tumor types (233, 234). Addressing this gap will require integrating functional validation into phenotypic and spatial studies, establishing standardized functional benchmarks, and determining whether Ttex accumulation represents immune failure, adaptive engagement, or context-dependent restraint.

### 8.3 Lack of longitudinal and dynamic tracking

T cell exhaustion is a dynamic and context-dependent process. However, most studies provide only static snapshots of Ttex phenotypes at a single time point (6, 235). There is a critical need for longitudinal studies that track the evolution of Ttex populations during disease progression, treatment response, and relapse. Techniques such as serial tumor biopsies, circulating T cell analysis, and T cell receptor (TCR) tracking can help elucidate the clonal stability and functional trajectory of Ttex in patients over time (43, 236). Future studies should incorporate longitudinal sampling and TCR-based lineage tracing in clinical trials to define Ttex stability, differentiation trajectories, and treatment-associated shifts, allowing more accurate prognostic and predictive modeling.

In addition, substantial methodological variability across studies, including differences in marker panels, spatial platforms, and computational thresholds, currently limits direct cross-study comparability and may contribute to apparent discrepancies in reported Ttex prognostic behavior.

### 8.4 Future research directions

Large-scale, prospective clinical studies are urgently needed to validate the prognostic and predictive value of Ttex and the Ttex/CD8^+^ ratio, ideally using standardized marker panels, unified scoring criteria, and longitudinal outcome tracking to establish clinically meaningful thresholds and applications (237). A central priority will be defining a universal Ttex marker panel anchored by PD-1, TOX, CD39, and loss of TCF1 to enable consistent identification across platforms and institutions. At the same time, integrating Ttex assessment into multi-omics immunoprofiling frameworks including genomics, transcriptomics, epigenetics, and spatial proteomics will deepen our understanding of the molecular programs driving terminal exhaustion and may further clarify the molecular programs driving terminal exhaustion and may reveal novel therapeutic vulnerabilities (238).

To support clinical translation, future work must establish tumor-type-specific Ttex/CD8^+^ ratio thresholds, validate digital pathology and AI-assisted pipelines across platforms, and develop harmonized, open-access benchmark datasets that ensure algorithmic reproducibility and regulatory readiness (239). Multi-center ring trials, standardized multiplex-staining calibration, and consensus reporting guidelines will be essential steps toward incorporating Ttex metrics into pathology workflows, risk-stratification frameworks, and clinical decision-support systems (240).

In summary, these gaps highlight that current conclusions regarding Ttex as biomarkers and therapeutic targets must remain provisional until validated by standardized, prospective, and mechanistically integrated clinical studies.

## 9 Conclusion

Ttex cells are increasingly recognized not merely as hallmarks of chronic antigen stimulation but as clinically informative immune populations that reflect the history, intensity, and organization of anti-tumor responses within the TME (8). While traditionally considered dysfunctional, Ttex retain residual effector potential and when evaluated in relation to total CD8^+^ T cell infiltration can serve as prognostic indicators in multiple solid tumor types (6).

Recent advances in multiplex imaging, spatial transcriptomics, and AI-driven digital pathology have made it feasible to quantify and map Ttex in clinical specimens, enabling translational integration into risk stratification, treatment planning, and companion diagnostics (88). Moreover, the technologies summarized in 2provide the methodological foundation for reliable detection and quantification of Ttex, supporting their incorporation into clinical workflows and translational research (158). The Ttex/CD8^+^ ratio emerges as a promising biomarker that captures both the quality and differentiation state of the tumor-infiltrating T cell compartment.

Nevertheless, important challenges remain. Inconsistent marker definitions limited functional validation, and a lack of longitudinal tracking hinder broader clinical adoption (5). Future efforts should prioritize the harmonization of assays and scoring systems to ensure reproducible detection across platforms. Large-scale, prospective studies across multiple cancer types will also be required to validate the prognostic and predictive value of Ttex in diverse clinical settings. Equally important is the integration of Ttex assessment into ongoing immunotherapy trials, which would refine patient stratification and therapeutic monitoring in real time (241).

In addition, deeper biological insights are likely to emerge from multi-omics approaches that combine spatial, transcriptomic, epigenetic, and metabolic profiling to define the molecular determinants driving the transition from Tpex to Ttex states (193). On the therapeutic side, strategies aimed at preventing or reprogramming terminal exhaustion including circRNA-based modulation, CRISPR-mediated editing, and rational checkpoint blockade combinations represent promising avenues to enhance T cell persistence and anti-tumor activity (187, 192).

Together, these future directions underscore the dual role of Ttex as both biomarkers and therapeutic entry points in precision immuno-oncology, highlighting their substantial promise for advancing immune monitoring and therapeutic innovation. By addressing the challenges in standardization, functional validation, and prospective clinical testing, the field can move closer to translating biological insights into robust, clinically actionable tools that improve outcomes for patients with solid tumors.
